# Recent Geological Events and Intrinsic Behavior Influence the Population Genetic Structure of the Chiru and Tibetan Gazelle on the Tibetan Plateau

**DOI:** 10.1371/journal.pone.0060712

**Published:** 2013-04-24

**Authors:** Fangfang Zhang, Zhigang Jiang, Aichun Xu, Yan Zeng, Chunwang Li

**Affiliations:** 1 Key Laboratory of Animal Ecology and Conservation Biology, Institute of Zoology, Chinese Academy of Sciences, Beijing, China; 2 Graduate School of the Chinese Academy of Sciences, Beijing, China; 3 School of Life Sciences, Jiliang University, Hangzhou, China; Natural History Museum of Denmark, University of Copenhagen, Denmark

## Abstract

The extent to which a species responds to environmental changes is mediated not only by extrinsic processes such as time and space, but also by species-specific ecology. The Qinghai-Tibetan Plateau uplifted approximately 3000 m and experienced at least four major glaciations during the Pleistocene epoch in the Quaternary Period. Consequently, the area experienced concurrent changes in geomorphological structure and climate. Two species, the Tibetan antelope (*Pantholops hodgsonii*, chiru) and Tibetan gazelle (*Procapra picticaudata*), both are endemic on the Qinghai-Tibetan Plateau, where their habitats overlap, but have different migratory behaviors: the chiru is inclined to have female-biased dispersal with a breeding migration during the calving season; in contrast, Tibetan gazelles are year-round residents and never migrate distantly. By using coalescence methods we compared mitochondrial control region DNA sequences and variation at nine microsatellite loci in these two species. Coalescent simulations indicate that the chiru and Tibetan gazelle do not share concordant patterns in their genealogies. The non-migratory Tibetan gazelle, that is more vulnerable to the impact of drastic geographic changes such as the elevation of the plateau, glaciations and so on, appears to have a strong population genetic structure with complicated demographic history. Specifically, the Tibetan gazelle population appears to have experienced isolation and divergence with population fluctuations since the Middle Pleistocene (0.781 Ma). However, it showed continued decline since the Upper Pleistocene (0.126 Ma), which may be attributed to the irreversible impact of increased human activities on the plateau. In contrast, the migratory chiru appears to have simply experienced population expansion. With substantial gene flow among regional populations, this species shows no historical population isolation and divergence. Thus, this study adds to many empirical studies that show historical and contemporary extrinsic and intrinsic processes shape the recent evolutionary history and population genetic structure of species.

## Introduction

Comparisons of genetic structure among sympatric species provide insights into the extent to which extrinsic and intrinsic factors interact to influence the geographic scale of population differentiation [1]–[4]. For example, ecologically and phylogenetically disparate taxa may exhibit striking concordance in phylogeographic structure across historical barriers [5]–[7]. Conversely, relatively minor differences in life history traits [8], [9] and ecology [10] among closely related species may translate into significant differences in the degree and scale of population structure.

In phylogeographic studies of animal taxa on the Qinghai-Tibetan Plateau, the relationship between environmental history and ecology is particularly pertinent as the plateau uplifted several times by approximately 3000 m during the Quaternary Period [11]. Furthermore, at least four major glaciations occurred in South-Central Asia during the Pleistocene [12], and widespread mountain glaciations once covered the Qinghai-Tibetan Plateau in the Lower Pleistocene epoch [13]. The uplift of the Qinghai-Tibetan Plateau and the associated or contemporaneous climate changes are widely regarded as the most important factors influencing current spatial distribution of local species and their genetic diversity on the plateau [14], [15]. In addition to the well-documented observation that population genetic structure is usually shaped by geographic and environmental factors [16], [17], some species-intrinsic behaviors and life history traits, for example, migration, dispersal and mating, can also affect the population genetic structure and recent evolutionary history of species [18]–[22].

The chiru and Tibetan gazelle primarily inhabit the Qinghai-Tibetan Plateau as sympatric bovines, but these species have distinct migrating behaviors. The chiru has wintering habitats and calving habitats. The female individuals migrate distantly (about 300 km) to the calving habitats in May and June each year to give birth to their calves, then they migrate back with their calves in early August to reunite with males in the wintering habitats [23]. In contrast, Tibetan gazelles only move around their habitats during their lifespan and never migrate distantly [24]. Given the similarities of the distribution on the plateau as well as the difference of their behaviors, these closely related species provide an excellent opportunity to study how extrinsic and intrinsic processes affect gene flow and population genetic structure.

Previous studies on the mtDNA haplotype data for the chiru and Tibetan gazelle suggested discordant demographic histories [25], [26]. Here we use mitochondrial control region sequence and highly polymorphic microsatellite variation to investigate population genetic structure, gene flow and genetic population history of the chiru and Tibetan gazelle. We first constructed population genetic structure and quantified population size by employing coalescent-based methods, then estimated gene flow rates and divergence times among populations. Specifically, by comparing the patterns of phylogeography and demography in the chiru and Tibetan gazelle species and taking advantage of the well-established geographical history of the Qinghai-Tibetan Plateau, we test the hypothesis that along with the environmental changes during the geological events that play important roles in phylogeography and genealogy for species, the intrinsic migratory behavior causes a differentiation in the population genetic structure of these two species.

## Materials and Methods

### Ethics statement

The chiru and Tibetan gazelle are listed in the Category I and Category II in the National Key Protected Wild Animal Species under the Wild Animal Protection Law of China respectively. Sample collection of these protected animals and field studies in Kekexili and other regions on the Qinghai-Tibetan Plateau adhered to the Wild Animals Protection Law of the People's Republic of China. All necessary permits were obtained for the described field studies.

### Sampling

**Figure 1:**
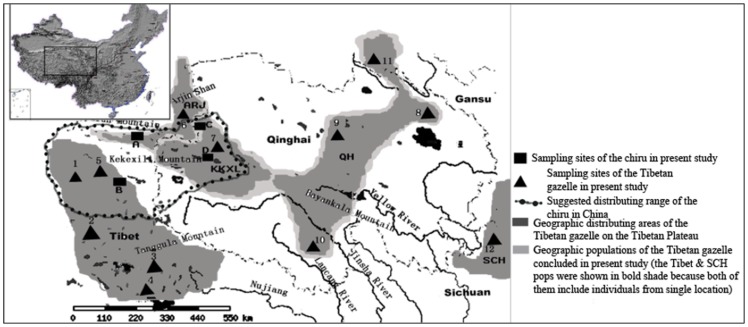
Map of the distribution and sampling locations. The sampling locations of the chiru (*Pantholops hodgsonii*) are indicated by rectangles (▪), and the capital letters indicate sampling locations (sample sizes in parentheses): A, Xinjiang (XJ, 19); B, Tibet (19); C, Qinghai (QH, 19); D, Zhuolaihu Lake (BH, 61). The sampling locations of the Tibetan gazelle (*Procapra picticaudata*) are indicated by black triangles (▴). The Arabic numerals indicate sampling locations (sample sizes in parentheses): 1, Geji (2); 2, Bange (2); 3, Mangkang (1); 4, Shengzha (1); 5, Qiangtang (4); 6, Arjin Shan (5); 7, Kekexili (8); 8, Tianjun (13); 9, Doulan (2); 10, Yushu (2); 11, Harshihar (1); and 12, Ruoergai (5).

Before the field expeditions, we obtained permission to conduct research and collect samples on the chiru and Tibetan gazelle from the Kekexili Nature Reserve, Hargai Nature Reserve, Changtang Nature Reserve and Arjinshan Nature Reserve. A total of 61 chiru muscle/skin samples were collected from the calving habitat of Zhuolaihu Lake, Kekexili, Qinghai Province (Fig. 1) in June and July 2005, when thousands of female chiru migrated there from wintering habitats to breed. The details of all the samples are listed in Table 1 and 2. All of the muscle and skin samples were collected from calves that died naturally. MtDNA sequences of the chiru from wintering habitats were derived from a previous [25] study (Table 1). We collected muscle and skin samples of the Tibetan gazelle from calves that died naturally or from the collections of several museums of different Nature Reserves; hair samples were from Tibetan gazelles in zoos or sick and/or injured ones rescued by the wardens of nature reserves, details are listed in Table 2. These samples represent the Tibetan gazelle populations through its distribution area on the Qinghai-Tibetan Plateau including Qinghai Province, Tibet Autonomous Region and adjacent Gansu Province, Sichuan Province, and Xinjiang Uigur Autonomous Region. A total of 51 samples of the Tibetan gazelle (Table 2) from five geographic populations at 12 sites (Fig. 1) were collected from February 2004 to September 2006.

**Table 1:** Sample sources, types and numbers for mtDNA and microsatellite analysis for chiru (*Pantholops hodgsonii*).

Region	Source	Type	mtDNA	msat^i^
Tibet	Derived from Ruan et al's study	*	19	/
Xinjiang, XJ	Derived from Ruan et al's study	*	19	/
Qinghai, QH	Derived from Ruan et al's study	*	19	/
Breeding habitat	Kekexili Nature Reserve	Skin, Muscle	61	51
Total			118	51

i Represents microsatellite; * No information; / No sample was analyzed.

**Table 2:** Sample information for mtDNA and microsatellite analysis for Tibetan gazelle (*Procapra picticaudata*).

Region	Source	Type	mtDNA	msat^i^
Kekexili, KKXL	Kekexili Nature Reseve	Skin, hair	8	8
Arjin, ARJ	Arjinshan Mountaion Nature Reserve	Skin	5	6
Tibet	Museum collection^#^	Skin, muscle	6	5
Tibet	The Forestry Bureau of Tibet	Skin	6	6
Tibet	Beijing Zoo	Hair	2	2
Sichuan, SCH	Ruoergai Nature Reserve	Skin	5	5
Qinghai, QH	The Forestry Bureau of Tianjun County	Muscle, hair	12	10
Qinghai, QH	Dulan International Hunting Park	Muscle	2	2
Qinghai, QH	Xining Zoo	Hair	3	3
Qinghai, QH	Harshihar International Hunting Park	Muscle	2	2
	Total		51	49

i Represents microsatellite; ^#^Samples from the Institute of Zoology, Chinese Academy of Sciences, representing three geographic locations.

### DNA extraction, PCR, DNA sequencing and genotyping

Total genomic DNA was extracted from the muscle, hair and skin samples using the standard proteinase K digestion and phenol/chloroform extraction procedures, after washing with excess NTE (0.05 M Tris–HCl, 0.01 M NaCl, 0.02 M EDTA, pH 8.0) to remove possible protease or PCR inhibitors. Approximately 580 bp of the mitochondrial DNA (mtDNA) control region was amplified from 51 Tibetan gazelle and 61 chiru samples. Forty-six samples of the Tibetan gazelle were used in a previous study; others are new to this study (Fig. 1). Primers, polymerase chain reaction (PCR) conditions and sequencing protocols were reported previously [26].

Nine microsatellite loci, originally isolated in cattle and pronghorn (*ETH225*, *ILSTS5*, *Aam7*, *CSSM43*, *TGLA122*, *BM1824*, *BM4107*, *BM1225*, *and BM1818*) [27]–[31], were amplified reliably and polymorphic in the two species. With one primer labeled by fluorochrome for each locus (FAM, HEX or TAMRA, Invitrogen), PCR amplifications were carried out in a volume of 15 µL using 2.0 mM MgCl_2_, 200 mM dNTPs, 0.4 mM of each primer, and 0.5 U of Taq DNA polymerase (Fergment). Amplified fragments were separated by capillary electrophoresis on an ABI PRISM 377-Avant Genetic Analyzer (Applied Biosystems). Allele sizes were determined relative to standard marker GeneScan-500 ROX (Applied Biosystems) using GENESCAN 3.7.

### Mitochondrial DNA sequence analysis

MtDNA control region sequences for all individuals were aligned using CLUSTAL X [32] and checked by eye. Intraspecific genetic diversity was estimated using haplotype diversity (*h*) and nucleotide diversity (π) as implemented in DnaSP version 4.0 [33].

#### Phylogeographic analysis

Genealogy of haplotypes within species were estimated using maximum-likelihood (ML), neighbor-joining (NJ) and maximum-parsimony (MP) by PAUP*4.0b8 [34] separately. The robustness of these analyses was assessed using bootstrap replications [35], with 1000 replications for MP and NJ and 100 replications for ML. In addition, Bayesian analysis was conducted using the Monte Carlo Markov chains (MCMC) method implemented in BEAST v1.7.2 [36]. We used a strict clock rate, with the substitution rate of 2×10^−8^ substitutions per site per year (S/S/year) [37]–[39]. Two replicates were run for 25 million generations with tree and parameter sampling every 1,000 generations. A burn-in of 10% was used and the convergence of all parameters was assessed using the software TRACER (within the BEAST package). The Bayes factor (BF) was used to assess alternative phylogenetic hypothesis in Bayesian framework (estimated in TRACER). A log (BF)>3 was considered positive support for one hypothesis versus another given the data [40]. Subsequently, the resulting samples under the BF-preferred model were summarized using the software TreeAnnotator using a posterior probability limit of 0.5, setting the height of each node in the tree to the median height across the entire sample of trees for that clad, and trees were visualized with FigTree [41]. The settings for the best-fit DNA substitution model were selected by the Akaike Information Criterion using MODELTEST 3.06 [42] and PAUP*.

Due to the low variation at the intraspecific level, traditional phylogenetic analyses often result in poorly resolved haplotype trees. In addition, coexistence of a persistent ancestral haplotype and its multiple descendants results in a haplotype tree with multifurcations [43]. Network approaches take these population-level phenomena into account, allowing more appropriate analysis of intraspecific data [44]. Network analysis was performed using the statistical parsimony algorithm implemented in TCS ver. 1.21 [45]. All sequences were included in the datasets to allow the calculation of haplotype frequencies.

Population pairwise *F*_ST_ and Φ_ST_ values for mtDNA were calculated using the program ARLEQUIN (version 2.0) [46]. Based on the best-fit models of sequence evolution for each species evaluated using MODELTEST above, Φ_ST_ was calculated using genetic distances estimated under the TVM model with specified gamma shape parameters for the chiru (α = 0.65), and under the HKY model with gamma distribution (α = 0.41) for the Tibetan gazelles. Because of the high proportion of unique haplotypes in the control region, estimates of population differentiation based on pairwise distances among haplotypes (Φ_ST_) [47] were more informative than differentiation estimates calculated from haplotype frequency (*F*_ST_). For this reason, we reported Φ_ST_ values only. The same program was used for analysis of molecular variance (AMOVA) [47] to test for differentiation between geographical populations within species. In AMOVA the correlation of pairwise distances is used as a Φ-statistic analog at various hierarchical levels. Φ_ST_ estimates the proportion of genetic variation within populations relative to the genetic variation for the whole sample, Φ_CT_ estimates the proportion of genetic variation among groups of populations relative to the whole species, and Φ_SC_ estimates the variation among populations relative to a regional grouping of populations. The significance of Φ-statistics was tested by random permutations of sequences among populations. The groupings that maximize values of Φ_CT_ and are statistically significant indicate the most parsimonious geographical subdivisions.

Furthermore, we used the program MIGRATE 3.1.6 [48], [49] to estimate maximum-likelihood migration rates among populations. This approach, based on coalescence using Markov chain Monte Carlo (MCMC) searches, takes account of unequally effective population sizes and asymmetrical gene flow [50]. Effective population size and gene flow rate were estimated from *F*_ST_ values and were set as initial values. We performed 10 short chains (500 trees used out of 10 000 sampled) and three long chains (5000 trees used out of 100 000 sampled). These runs were repeated using the same condition until consistent results were obtained.

#### Estimate of demography

Historical population dynamics of the species were estimated using coalescent-based Bayesian skyline plots (BSP) [51] as well as mismatch distributions [52]. BSPs were implemented in BEAST. They were used to estimate the dynamics of past populations through time without requiring a pre-specified parametric model of demographic history. Uncertainty in the genealogy was controlled using a Bayesian approach under a coalescence model. The Bayesian Skyline Plot model with a strict clock was selected to construct the BSP in BEAST for each species. Chains were run for 10 million generations, sampled every 1000 generations and the first 10% of the trees were discarded as burn-in. The results were summarized using Tracer. Mismatch distributions were calculated using ARLEQUIN. Multimodal distributions were expected in populations at demographic equilibrium or in decline, and unimodal distributions were anticipated in populations having experienced a recent demographic expansion [53], [54]. The expected distributions were generated by bootstrap resampling (10,000 replicates) using a model of sudden demographic expansion. The sum of square deviations and raggedness index between the observed and the expected mismatch were used as test statistics. P-values were calculated as the probability of simulations producing a greater value than the observed value. In addition, we chose two test statistics to test whether two data sets conform to expectations of neutrality, each with particular sensitivity to one demographic scenario. Fu and Li's D* is designed to detect an excess of old mutations, characteristics of a population that has experienced a historical reduction in effective population size [55], [56]. In contrast, Fu's *Fs* is sensitive to an excess of recent mutations, a pattern typical to both demographic expansion and selective sweep [57], [58]. Fu and Li's D* was calculated in DNASP (version 4.0), Fu's *Fs* differences were tested for significance with a coalescent simulation program (1000 simulations), as implemented in ARLEQUIN 2.000.

#### Isolation by distance

Mantel tests were employed to determine whether significant isolation-by-distance exists among populations by testing for correlation between pairwise Φ_ST_ values and geographic distance using the Isolation-by-Distance Web Service 3.16 [59]. Mantel tests were performed with 20,000 iterations that included negative ΦST values and again with negative Φ_ST_ values converted to zeros.

### Microsatellites analysis

Intraspecific allelic richness and heterozygosity were calculated in FSTAT (version 2.9.3.2) [60]. Deviations from Hardy-Weinberg Equilibrium, heterozygote deficits and linkage equilibrium were tested in GENEPOP [61].

The software STRUCTURE version 2.3.3 [62] was used to evaluate the potential substructure of the two species by estimating the number of subpopulations (*K*). Population numbers *K* = 1–7 were tested for 20 times at the population level based on 100,000 generations (MCMC) after a burn-in period of 10,000. For all STRUCTURE simulation runs we used the admixture model and the independent allele frequencies model, either with or without the location prior model, and set all other run parameters to their default values. Because it is reported that in some cases the number of *K* estimated by structure does not correspond to the real number of subpopulations [63]. The Δ*K* rates of change of Ln P (D) (estimated log probability of data) for *K* inferred clusters were analyzed here. To display STRUCTURE Q plots, DISTRUCT [64] was used to generate color-coded bar graphs for the Tibetan gazelle (Because the chiru samples for STR statistics were from breeding habitat only, this analysis was not conducted to the chiru).

The program BOTTLENECK (version 1.2.02) [65] was used to detect potential recent bottlenecks in each species. Analyses can be run assuming an infinite alleles model (IAM), a stepwise-mutation (SMM) or a two-phase model (TPM), which incorporates a user-specified proportion of SMM into a multistep mutation model. We ran analyses under the IAM, SMM and TPM (with 30% SMM). Significant departures from the heterozygosity expectations estimated under a given mutation model reject a null hypothesis of mutation-drift equilibrium. A significant excess or deficit in heterozygosity is interpreted as evidence for a demographic expansion or contraction, respectively [65]. The rationale for these expectations is that following a significant reduction in effective population size, the observed number of alleles in a population will be less than that expected from the expected heterozygosity. Conversely, following a significant increase in effective population size, the observed number of alleles is expected to exceed that predicted from expected heterozygosity.

## Results

### Diversity indices

For mitochondrial control region DNA sequence, a total of 124 variable nucleotide sites were observed in the chiru, of which 76 were parsimony-informative, which defined 86 haplotypes (50 haplotypes were derived from GenBank, GenBank accession Nos. **AY744081**–**AY744130**; other 36 haplotypes were obtained in present study, GenBank accession Nos. **JQ292928**–**JQ292963**). For the Tibetan gazelle, 193 variable nucleotide sites were observed, and 130 were parsimony-informative, which defined 25 haplotypes (GenBank accession Nos. **DQ017352**–**DQ017355** and **DQ423488**–**DQ423508**). For both species, the haplotype diversity was about the same (0.975–0.991), but the nucleotide diversity of the Tibetan gazelle was 4.2 times that of the chiru (Table 3).

**Table 3:** Sample sizes (*n*) and diversity indices for the chiru (*Pantholops hodgsonii*) and Tibetan gazelle (*Procapra picticaudata*).

Species	mtDNA						Microsatellites			
	n	haplotype	π	*h*	*Fs*(p value)	D*(p value)	n	*k*	*H*_E_	*H*_O_
chiru	118	86	0.025±0.10	0.991±0.003	−24.08 (<0.001)	−2.660 (>0.1)	51	94	0.819	0.792
Tibetan gazelle	51	25	0.105±0.01	0.975±0.014	0.971 (0.712)	0.377 (>0.1)	49	115	0.789	0.752
										

Total number of haplotypes per species, mean nucleotide diversity (*π*), mean haplotype diversity (*h*), Fu's *Fs* and Fu and Li's D* were from mtDNA sequences; total number of alleles per species (*k*), expected heterozygosity (*H*_E_) and observed heterozygosity (*H*_O_) were based on nine nuclear microsatellite loci.

For microsatellite loci, linkage disequilibrium was detected between one pair of the loci in the chiru, but no linkage disequilibrium was detected among any of the loci in Tibetan gazelles. Several departures from Hardy-Weinberg Equilibrium were detected; two loci (BM1225 and BM1818) showed significant heterozygosity deficit in both species. Observed heterozygosity was 0.792 and 0.752 (Table 3) in the chiru and Tibetan gazelles respectively, and alleles per polymorphic locus ranged from 6 to 20.

### Haplotype phylogenetic relationships

**Figure 2:**
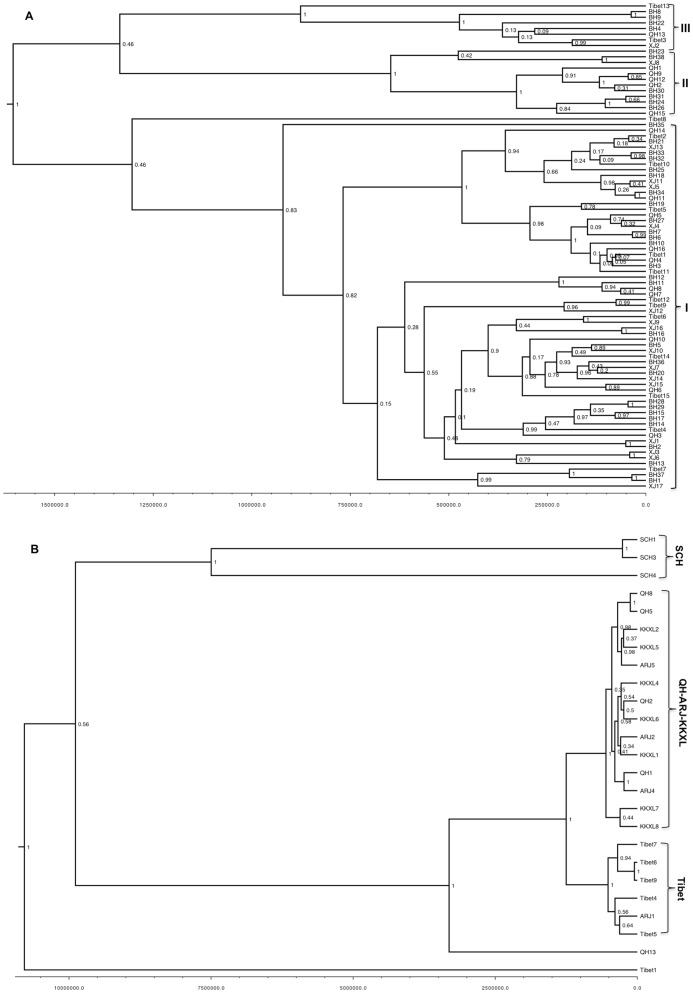
Phylogenetic relationships. Bayesian inferred (BI) trees among the mitochondrial control region haplotypes for A) chiru (*Pantholops hodgsonii*), and B) Tibetan gazelle (*Procapra picticaudata*). Posterior possibilities are indicated next to nodes.

**Figure 3:**
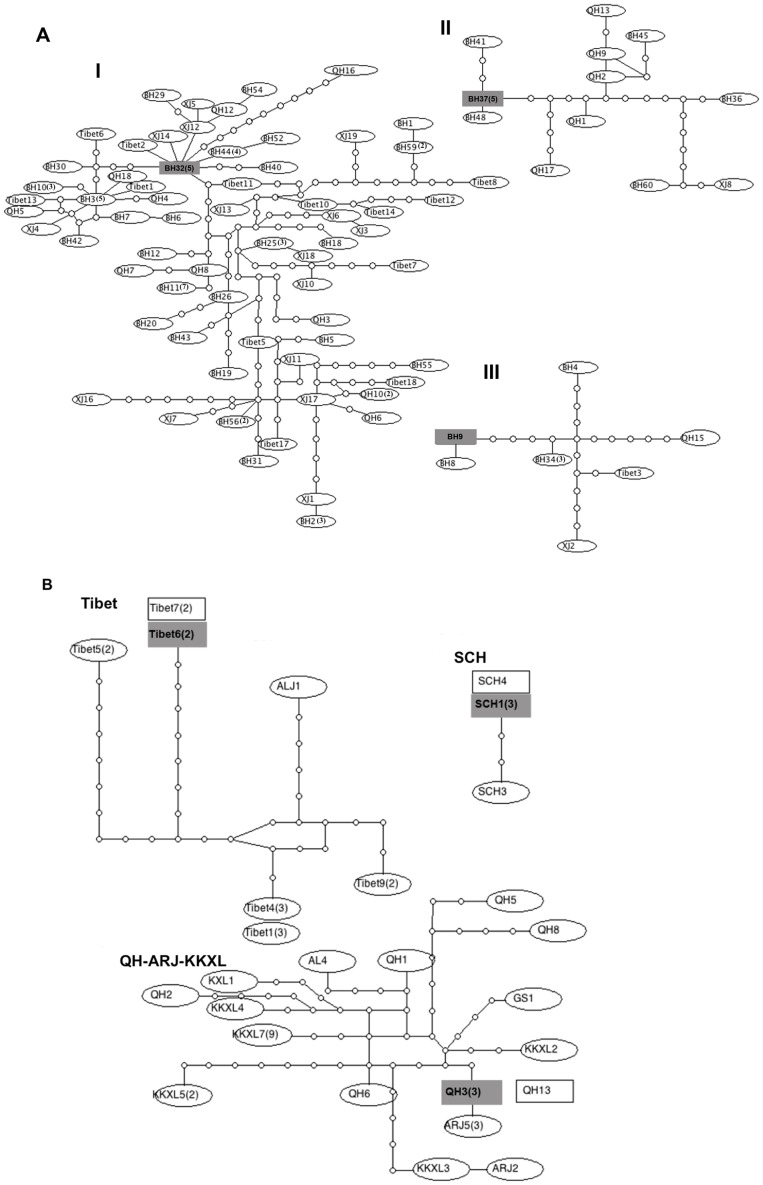
Haplotype networks. TCS generated haplotype networks of A) chiru (*Pantholops hodgsonii*), and B) Tibetan gazelle (*Procapra picticaudata*) based on the mitochondrial DNA sequences. Numbers in the parentheses denote the haplotype frequencies.

The gene tree topologies from maximum likelihood and Bayesian Inference (using BEAST) analyses are identical. The BF strongly favored the constant population model for the chiru (log (BF)>6), but positively supported Bayesian Skyline model for the Tibetan gazelle (log (BF)>3). Thus, Bayesian Inference analyses were performed under these models respectively. For the chiru, no strong geographic structure was inferred by the phylogenetic analysis of mtDNA (Fig. 2A), and the basal placement of haplotypes into three major clades suggests a pattern of population divergence. While one of the clades (clade I) showed very high posterior probability (posterior probability = 1, Fig. 2A), the posterior probability didn't support the other clades (posterior probability = 0.46, Fig. 2A). In addition, all the three clades were either composed of haplotypes from wintering and calving habitats or mixed haplotypes from different wintering and calving habitats. Furthermore, the genetic network analysis of the mtDNA sequences of the chiru coincides with the haplotype tree patterns. Although TCS analysis resulted in three unconnected networks using statistical parsimony with a 95% confidence limit (Fig. 3A), these three independent clades (clade I, II and III) lack of clear geographic patterns as each of the clades includes chiru individuals from different geographic locations. Discordantly, the 25 Tibetan gazelle haplotypes showed three major clusters: Tibet, SCH and QH-ARJ-KKXL (Fig. 2B). The SCH cluster was formed with a moderate posterior probability (posterior probability = 0.56), but the Tibet and QH-ARJ-KKXL clusters showed very high posterior probability support (posterior probability = 1 respectively). In addition, the genetic network analysis of the mtDNA sequences connected Tibetan gazelles into three main unconnected networks under the 95% statistical parsimony criterion of TCS (Fig. 3B). Populations corresponding to [SCH], [Tibet] and [QH, ARJ and KKXL] were three independently connected networks. Four haplotypes were outliers in the network.

### Population structure and gene flow

Pairwise Φ_ST_ statistics of the mtDNA sequences showed no apparent subdivision in the chiru (Table 4). AMOVA indicated no significant Φ_CT_ value in possible population groupings. The genetic variation was explained by variation within populations relative to the whole sample (Φ_ST_; Table 5). Consistently, significant level of historical gene flow was detected from 5/6 possible source-recipient relationships between pairs of regional groups (Table 6). Conversely, for the Tibetan gazelle, the genetic differentiation was detected and significant between the SCH and each of the other four regional populations, as well as the Tibet versus the other three regional populations (excluding ARJ) in pairwise Φ_ST_ values (Table 7). Analysis of AMOVA indicated a single significant Φ_CT_ value in one of the possible population grouping patterns: Tibet, SCH and QH-ARJ-KKXL (Table 8, Fig. 1). Significant difference (*p*<0.01; Table 8, 3000 permutations) among the three groups was observed. In addition, this grouping pattern gave the highest Φ_CT_ value (0.0676). Consistently, no significant historical gene flow was detected from most of the possible source-recipient relationships between pairs of the regional Tibetan gazelle populations (14/20, Table 9). In addition, no significant gene flow was found in the six possible source-recipient relationships between pairs of phylogeographic groups inferred from pairwise Φ_ST_ statistics (Tibet, SCH and QH-ARJ-KKXL, Table 10).

**Table 4:** Pairwise population differentiation values and Φ_ST_ values for chiru (*Pantholops hodgsonii*).

	Tibet	XJ	QH
Tibet	17.41	−0.017	0.022
XJ	0.79	16.40	0.053
QH	0.16	0.091	21.98

Above diagonal: Pairwise Φ_ST_ values between populations. Diagonal elements: Average number of pairwise differences within population (PiX). Below diagonal: Corrected average pairwise difference (PiXY−(PiX+PiY)/2). Pairwise Φ_ST_ values and corrected average pairwise differences that are statistically different are indicated.

**Table 5:** AMOVA for grouping of populations estimated using Φ-statistics based on control region sequence for chiru (*Pantholops hodgsonii*).

Groups	Among pops within groups (Φ_SC_)	Within pops (Φ_ST_)	Among groups (Φ_CT_)	% Among groups	*P* (Among groups)
[Tibet] [QH & XJ]	0.0557	0.0026	−0.0562	0	1.000
[QH] [Tibet & XJ]	−0.0205	0.0410	0.0603	6	0.8299
[XJ] [Tibet & QH]	0.0259	0.0180	−0.0081	0	0.3314

**Table 6:** Estimates of gene flow (Nem) and theta between regional groups of chiru (*Pantholops hodgsonii)*.

Population (x)	Theta (2Neµ)	Values of 2 Nm [x = receiving population]		
		XJ, x	Tibet, x	QH, x
XJ	0.06094	–	4.8418	131.9213
Tibet	0.16855	256.5377	–	141.3462
QH	0.08069	699.7087	1050.9922	–

Ne is the effective population size of females, µ is the mutation rate and m is the migration rate.

**Table 7:** Pairwise population differentiation values and Φ_ST_ values for Tibetan gazelle (*Procapra picticaudata*).

	Tibet	ARJ	SCH	KKXL	QH
Tibet	61.29	−0.144	0.5384**	0.262**	0.422**
ARJ	−6.33	56.67	0.546**	0.057	0.233
SCH	46.35**	46.83**	49.67	0.742**	0.830**
KKXL	16.91*	0.22	66.67**	7.40	−0.027
QH	16.09**	0.02	65.09**	0.03	10.84

* *p*<0.05.

** *p*<0.01.

Above diagonal: Pairwise Φ_ST_ values between populations. Diagonal elements: Average number of pairwise differences within population (PiX). Below diagonal: Corrected average pairwise difference (PiXY−(PiX+PiY)/2). Pairwise Φ_ST_ values and corrected average pairwise differences that are statistically different are indicated.

**Table 8:** AMOVA for grouping of populations estimated using Φ-statistics based on control region sequence for Tibetan gazelle (*Procapra picticaudata*).

Groups	Among pops within groups (Φ_SC_)	Within pops (Φ_ST_)	Among groups (Φ_CT_)	% Among groups	*P* (Among groups)
[Tibet] [QH, KKXL, ARJ&SCH]	0.0494	0.0812	0.0335	3	0.1906
[Tibet] [QH, KKXL, ARJ] [SCH]	0.0190	0.0854	0.0676**	7	<0.01
[Tibet, QH, KKXL, ARJ] [SCH]	0.0475	0.1027	0.0579	6	0.1945
[Tibet, KKXL, ARJ] [QH] [SCH]	0.0139	0.0750	0.0620	6	0.0968
[Tibet, KKXL] [ARJ, QH] [SCH]	0.0503	0.0673	0.0179	2	0.0626
[Tibet, SCH] [ARJ, KKXL, QH]	0.0538	0.0704	0.0176	2	0.1046

** Significant at 0.01 level.

**Table 9:** Estimates of gene flow (Nem) and theta between regional groups of Tibetan gazelle (*Procapra picticaudata*).

Population (x)	Theta(2Neµ)	Values of 2 Nm [x = receiving population]				
		Tibet, x	SCH, x	ARJ, x	KKXL, x	QH, x
Tibet	0.05889	–	1.07e−10	2.83e−07	9.8061	3.44e−08
SCH	0.10051	1.25e−13	–	9.36e−14	9.40e−14	3.4915
ARJ	0.03743	47.4050	9.36e−14	–	76.4164	52.7836
KKXL	7.29e12	1.07e−06	4.15e−09	1.69e−13	–	3886.2540
QH	0.01132	9.36e−14	5.65e−10	1613.2711	3233.7368	–

Ne is the effective population size of females, µ is the mutation rate and m is the migration rate.

**Table 10:** Estimates of gene flow (Nem) and theta between geographic groups of Tibetan gazelle (*Procapra picticaudata*).

Population (x)	Theta(2 Neµ)	Values of 2 Nm [x = receiving population]		
		Tibet, x	Sichuan, x	QRK, x
Tibet	0.04466	–	1.27e−12	5.3019
SCH	0.10730	2.8868	–	5.13e−08
QH-ARJ-KKXL	0.06506	16.0895	7.82e−10	–

Ne is the effective population size of females, µ is the mutation rate and m is the migration rate.

**Figure 4:**
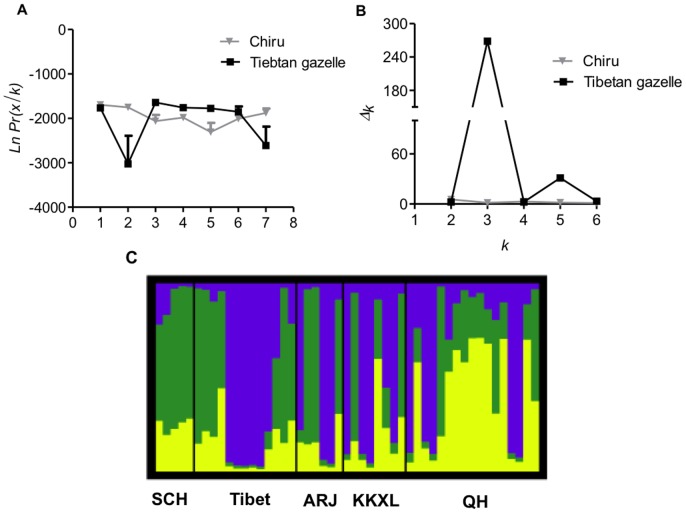
*K*, delta *K* scores and Q-plot. A) & B) *K* and delta *K* scores of the chiru (*Pantholops hodgsonii*) and Tibetan gazelle (*Procapra picticaudata*). The scores are based on microsatellite data with all loci included and prior assumptions of 1–7 genotypic clusters (*K*); C) Q-plot of the Tibetan gazelle (*Procapra picticaudata*) at *K* = 3.

Likewise, in microsatellite data set of the chiru, no evidence of population structure was detected in the Bayesian clustering analysis (with no prior population assignments) with the highest likelihood score at *K* = 1(Fig. 4A). Consistently, the delta *K* plot of the chiru showed no clear peak for *K* = 2–7 (Fig. 4B). However, for the Tibetan gazelle, Bayesian clustering analysis of the microsatellite variation inferred genetically distinct groups (between two and four). The peak likelihood score was at *K* = 3, but were substantially worse at *K* = 2 and *K* = 4 (Fig. 4A). Furthermore, the Δ*K* showed a significant peak exactly at *K* = 3 (Fig. 4B). Based on these results, we estimated the Q-plot of the Tibetan gazelle for *K* = 3 (Fig. 4C). The Q-plot for *K* = 3 inferred that the Tibetan gazelles from SCH corresponded to the cluster1, the individuals from Tibet corresponded to the cluster2, and the majority of the gazelles from Qinghai (QH), Arjinshan (ARJ) and Kekexili (KKXL) corresponded to the cluster 3, which was consistent with the mtDNA results.

No significant positive correlation between genetic and geographic distance (isolation by distance, IBD) was observed either among the chiru (Mantel test; r = −0.333, p = 0.30) or Tibetan gazelle populations (Mantel test; r = −0.347, p = 0.21) in mtDNA sequence data. The results indicate that the different population structure between these two species was not explained by geographic distance.

### Demographic analysis

**Figure 5:**
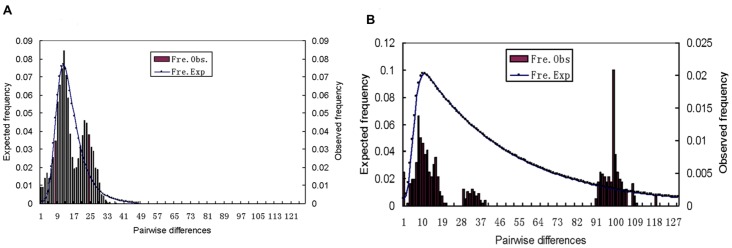
Mismatch distributions. Mismatch distributions of mtDNA control region for A) chiru (*Pantholops hodgsonii*) and B) Tibetan gazelle (*Procapra picticaudata*). The parameters of the goodness-of-fit test to the model of sudden expansion [52] are: sum of squared deviations (SSD), 0.0049 for the chiru (*p* = 0.37), and 0.0239 for the Tibetan gazelle (*p* = 0.224); r, raggedness index, 0.0024 for the chiru (*p* = 0.6), and 0.0216 for the Tibetan gazelle (*p* = 0.047).

**Figure 6:**
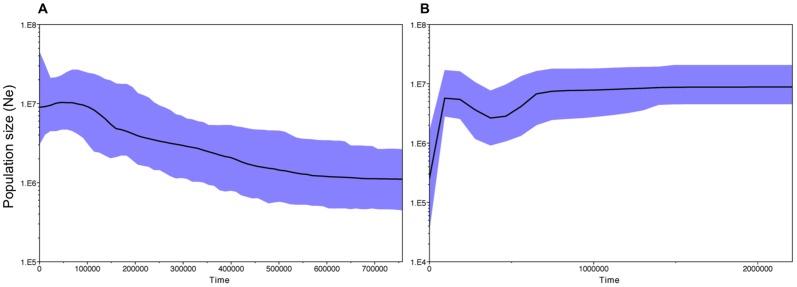
Bayesian skyline plots. Bayesian skyline plots showing the demographic history of A) chiru (*Pantholops hodgsonii*) and B) Tibetan gazelle (*Procapra picticaudata*). Dark lines represent median values for the population size (*Ne*); blue area marks the 95% highest probability density intervals in all panels.

While the mismatch distribution of mtDNA was not unimodal for the chiru, the accumulation of low-frequency mutations was characteristic of nonequilibrium population dynamics (Fig. 5A). In addition, the sum of square deviations and raggedness index suggested no significant difference between the observed distribution and the distribution expected under a model of sudden demographic expansion. Further, Fu's *Fs* test detected highly significant departures from the neutral/equilibrium expectations (p<0.001). Fu and Li's D* test showed result of no significance (Table 3). Consistently, 8/9 and 6/9 microsatellite loci showed heterozygote excess under the model IAM and TPM respectively, which also suggests a recent population expansion of the chiru (data not show). Further, the BSP analysis showed support for the hypothesis of population growth of the chiru during the Pleistocene (Fig. 6A), although the Bayesian Factor (BF) favored the constant population model for this species. In contrast, for the Tibetan gazelle, the shape of the mismatch distribution derived from the mtDNA was ragged and multimodel, suggesting a long-term demographic stability or declining demography (Fig. 5B). Consistently, nonsignificant values for both Fu's *Fs* (p = 0.712) and Fu and Li's D* indicated that the sequence evolution of the Tibetan gazelle is consistent with the expectation of selective neutrality and stable demographic history. Results of the test for demographic fluctuation based on microsatellite heterozygosities of the Tibetan gazelle were nonsignificant under the IAM, SMM and TPM, and the microsatellite allele frequency distribution was L-shaped (data not shown), suggesting no obvious population expansion or bottleneck [66], [67]. In addition, with the positive support by the BF, BSP analysis of the Tibetan gazelle provides further details. Although there were population fluctuations, the demographic trend of this species appears to remain relatively stable before the Upper Pleistocene (0.126 Ma). But after that, surprisingly, the population size of the Tibetan gazelle began to decrease sharply (Fig. 6B).

## Discussion

Overall agreement between genetic data and ecological associations indicates that genetic differentiation corresponds to biologically meaningful differences in environment and ecology (behavior). The deep level of population genetic structure in the Tibetan gazelle contrasts with the shallow divergence in the chiru, indicating the discordance of the population structure between these two sympatric species on the Tibetan Plateau. Although high haplotype diversity was detected both in the chiru and the Tibetan gazelle populations (Table 3), more population differentiation was found in the Tibetan gazelle (average Φ_ST_ = 0.363 with 6/10 significantly differentiated population comparisons), comparing to that of the chiru (average Φ_ST_ = 0.019 with no significantly differentiated population comparison). In addition, the Tibetan gazelle exhibits a stronger pattern of isolation between geographic populations (a significant Φ_CT_ was found in AMOVA) than the chiru, but it seems this isolation is not related to the distance.

Whether the historical environmental changes have a differential or overriding impact on the population depends on the complicated interaction between various factors. Large sequence divergences have been reported, for example, among divergent mtDNA genotypes separated by geographical barriers or distance [68] or within hybrid zones [69], [70]. In present study, IBD analyses showed no significant positive correlation between genetic and geographic distance both in the chiru and Tibetan gazelle. Thus the phylogeographic divergence between the populations of the Tibetan gazelle may be related to geographic barriers. For example, the Kunlun Mountains, Tanggula Mountains, Lanchang River, Nujiang River, Jinshajiang River, Qionglai Mountains and Daxueshan Mountains are all natural barriers that separate the Tibet, Qinghai and Sichuan populations of the Tibetan gazelle. Furthermore, the population divergence of the Tibetan gazelle is consistent with the historical environmental changes of the Qinghai-Tibetan Plateau. According to previous studies, the substitution rate for the mammal mitochondrial control region (CR) sequence is 2–4% per million years [37]–[39], and based on the estimated divergence rate of the CR (average sequence divergence 15.4%, results not shown), a recent coalescence time of approximately 3.85–7.7 million years was predicted among the Tibetan gazelle samples, which matches the conclusion by An et al. [71] from their geographical study that enhanced uplift of the Qinghai-Tibetan Plateau along the northern and eastern margins occurred 3.6–2.6 million years ago. Similarly, it was reported that the largest bovid living on the Qinghai-Tibetan Plateau, Yak (*Bos grunnience*), and domestic cattle were estimated to have diverged approximately 4.9 million years ago [72]. Thus, this enhanced uplift of the Qinghai-Tibetan Plateau could play an important role in the speciation of endemic species on the Plateau and divergence of the Tibetan gazelle populations.

In contrast, phylogeographic analyses for the chiru, even with haplotypes from all regions including the wintering habitats and calving habitat, indicate spatial homogeneity in mtDNA sequence variation, which is comparable to the findings in other migratory species [73]. Because genetic homogeneity typically implies sufficient gene flow (migration) to offset genetic divergence, it has generally been hypothesized [74], [75] that extensive movement likely occurs during one or more life-history stages. Field investigations into the migratory population of the chiru have found breeding associated migration in all geographic populations [76], [77]. During the course of migration for calving, it is possible that a number of individual females and their calves migrate to other wintering habitats instead of their original locations [78], thereby helping to promote gene exchange between populations of different localities. The frequent and high matrilineal gene flow (the highest was 1051) between pairs of regional populations implies further explanation for genetic homogeneity of the chiru.

Despite the high overlap in current habitat associations and geographic distribution, demographic analyses for these two species demonstrate different population histories. BSP results suggested that the population size of the Tibetan gazelle was relatively constant over a long period of time, even during the periods with extensive geographic changes (the elevation of the plateau) and glaciations (Fig. 6B). This may be attributed to the topographical diversity on the Qinghai-Tibetan Plateau that created networks for refugia for wild animals during glaciations [79]. However, surprisingly, the population of the Tibetan gazelle began to decrease sharply around the Upper Pleistocene Period. A previous report of population decrease in bison [80] around 37,000 years ago suggested that ecological changes might have been sufficient to affect this large mammal and stress its population. But Stiller et. al [81] found that the extinction of the cave bear about 24,000 years ago was unlikely to have been driven entirely by the climatic changes of the Last Glacial Maximum. Instead, it is possible that modern human activities (direct hunting or cave competition) were responsible for that. These studies support the possibility that the combination of environmental changes and human activities could have caused the population decline of the Tibetan gazelles. The topographical diversity of the Qinghai-Tibetan Plateau might have created complex barriers for these species to expand. On the other hand, the appearing and increase of nomadic activities on the plateau [82], for example, the road and railway construction, hunting as well as other human activities restrict the gazelles into separated patches. Consequently, the disruption of the evolutionary processes [83] can easily cause the decrease of the population size of the non-migratory Tibetan gazelle. Similar population history was reported on the demographic analyses of the Przewalski's gazelle, another similar species to the Tibetan gazelle. The Przewalski's gazelle, that habitats the eastern part of Qinghai-Tibetan Plateau, has experienced a genetic bottleneck and severe population decline, with effective population size reduced to less than one percent mainly due to the human activities [84].

However, the chiru showed a simpler demographic history. The shape of the mismatch distribution is generally in good agreement with a model of population growth. Although Bayesian Factor showed no support for the hypothesis, the Bayesian skyline plot demonstrated that the population size of the chiru has been in a pattern of growth. But it was slightly suppressed during the Upper Pleistocene Period (around 45,000 years ago), which may lend support for the constant population hypothesis suggested by BF. This slight population decrease could also be the result of both environmental changes (geographic and climate changes) as well as increased human activities. Migration, the significant characteristics of the chiru, is clearly required for successful breeding. But during the glacial periods, the ice sheets could make the migration difficult or even impossible, which could limit the breeding behavior. Also, increased human activities and constructions [82] may limit the breeding migration of the chiru. Thus, the effective breeding population size will decrease, which eventually can lead to the contraction of the whole population. On the other hand, the migration behavior can keep sufficient gene flow after the retreat of the glaciations, which can counteract the negative impacts from the environment and human beings, and keep a constant population size or even result in a population expansion.

Our study underscores the intrinsic and extrinsic roles played by the elevation of the Tibetan Plateau, glaciations as well as migration in shaping the patterns of the genetic structure and demography among closely related species. For the Tibetan gazelle, historical separation and the absence of gene flow between localities would be expected, in time, to lead to heterogeneity of mtDNA haplotypes [85]. But for the chiru, although some factors such as glaciations and human activities affected the population slightly, the migration behavior may offset the population isolation and divergence, and even plays an important role in the maintaining and recovery of the population. Similar historical demography was found in the migratory and non-migratory Mexico free-tailed bat groups due to gender-biased migratory behavior [86]. The present study highlights the value of comparative analyses of closely related sympatric species, and the importance of considering multiple aspects of species ecology when developing and testing phylogeographic and demographic history hypotheses.

## References

[1] Bermingham E, Moritz C (1998) Comparative phylogeography: concepts and applications. Mol Ecol 7: 367–369.

[2] Avise JC (2000) Phylogeography: The History and Formation of Species. Cambridge (Massachusetts) : Harvard University Press.

[3] Patton JL, Silva Da MNF, Malcolm JR (2000) Mammals of the Rio Jurua and the evolutionary and ecological diversification of Amazonia. B Am Mus Na Hist 244: 13–306.

[4] Arbogast BS, Kenagy GJ (2001) Comparative phylogeography as an integrative approach to historical biogeography. J Biogeogr 28: 819–825.

[5] Joseph L, Moritz C, Hugall A (1995) Molecular support for vicariance as a source of diversity in rainforest. P Roy Soc Lond B Bio 260: 177–182. 10.1098/rspb.1995.0077 7784437

[6] Schneider CJ, Cunningham M, Moritz C (1998) Comparative phylogeography and the history of endemic vertebrates in the wet tropics rainforests of Australia. Mol Ecol 7: 487–498.

[7] Evans JB, Supriatna J, Andayani N, Setiadi MI, Cannatella DC, et al (2003) Monkeys and toads define areas of endemism on the island of Sulawesi. Evolution 57: 1436–1443. 12894950 10.1554/02-443

[8] Patton JL, Silva Da MNF, Malcolm JR (1996) Hierarchical genetic structure and gene flow in three sympatric species of Amazonian rodents. Mol Ecol 5: 229–238. 8673269 10.1111/j.1365-294x.1996.tb00310.x

[9] Matocq MD, Patton JL, Silva Da MNF (2000) Population genetic structure of two ecologically distinct Amazonian spiny rats: separating history and current ecology. Evolution 54: 1423–1432. 11005308 10.1111/j.0014-3820.2000.tb00574.x

[10] Brouat C, Sennedot F, Audiot P, Leblois R, Rasplus J-Y (2003) Fine-scale genetic structure of two carabid species with contrasted levels of habitat specialization. Mol Ecol 12: 1731–1745. 12803627 10.1046/j.1365-294x.2003.01861.x

[11] Zhang D (2000) Eco-environmental effects of the Qinghai-Tibet Plateau uplift during the Quaternary in China. Environ Geol 39: 1352–1358.

[12] Chen Y (1984) Quaternary climates. Shijiazhuang: Hebei Normal University Press.

[13] Kwan HK, Choung ML, Liu T, Ding M, Derbyshire E (1996) Gravel deposits on the margins of the Qinghai-Xizang (Tibetan) Plateau, and their environment significance. Palaeogeography, Palaeoclimatology, Palaeoecology 120: 159–170.

[14] Fort M (1996) Late Cenozoic environmental changes and uplift on the northern side of the central Himalaya: a reappraisal from field data. Palaeogeography,Palaeoclimatology, Palaeoecolog 120: 123–145.

[15] Hewitt G (2000) The genetic legacy of the Quaternary ice age. Nature 405: 907–913. 10879524 10.1038/35016000

[16] Manel S, Schwartz MK, Luikart G, Taberlet P (2003) Landscape genetics: combining landscape ecology and population genetics. Trends Ecol Evol 18: 189–197.

[17] Coulon A, Guillot G, Cosson JF, Angibault JMA, Aulagnier S, et al (2006) Genetic structure is influenced by landscape features: empirical evidence from a roe deer population. Mol Ecol 15: 1669–1679. 16629819 10.1111/j.1365-294X.2006.02861.x

[18] Palumbi SR, Baker CS (1994) Contrasting population structure from nuclear intron sequences and mtDNA of humpback whales. Mol Bio Evol 11: 426–435. 7912407 10.1093/oxfordjournals.molbev.a040115

[19] Petit E, Balloux F, Goudet J (2001) Sex-biased dispersal in a migratory bat: a characterization using sex-specific demographic parameters. Evolution 55: 635–640. 11327171 10.1554/0014-3820(2001)055[0635:sbdiam]2.0.co;2

[20] Aboim MA, Menezes GM, Schlitt T, Rogers AD (2005) Genetic structure and history of populations of the deep-sea fish Helicolenus dactylopterus (*Delaroche*, 1809) inferred from mtDNA sequence analysis. Mol Ecol 14: 1343–1354. 15813775 10.1111/j.1365-294X.2005.02518.x

[21] Rivers NM, Butlin RK, Altringham JD (2005) Genetic population structure of Natterer's bats explained by mating at swarming sites and philopatry. Mol Ecol 14: 4299–4312. 16313594 10.1111/j.1365-294X.2005.02748.x

[22] Reddy PA, Gour DS, Bhavanishankar M, Jaggi K, Hussain SM, et al (2012) Genetic Evidence of Tiger Population Structure and Migration within an Isolated and Fragmented Landscape in Northwest India. PLoS ONE 7(1): e29827 doi:10.1371/journal.pone.0029827. 22253791 10.1371/journal.pone.0029827 PMC3256177

[23] Schaller GB (1998) Wildlife of the Tibetan Steppe. Chicago: The University of Chicago Press.

[24] Hu J, Jiang Z (2012) Detecting the potential sympatric range and niche divergence between Asian endemic ungulates of *Procapra* . Naturwissenschaften 99: 553–565. 22743804 10.1007/s00114-012-0933-1

[25] Ruan X, He P, Zhang J, Wan Q, Fang S (2005) Evolutionary history and current population relationships of the chiru (*Pantholops hodgsonii*) inferred from mtDNA variation. J Mammal 86: 881–886.

[26] Zhang F, Jiang Z (2006) Mitochondrial phylogeography and genetic diversity of Tibetan gazelle (Procapra picticaudata): implications for conservation. Mol Phylogenet Evol 41: 313–321. 16837214 10.1016/j.ympev.2006.05.024

[27] Brezinsky LS, Kemp J, Teale AJ (1993) ILSTS005: a polymorphic bovine microsatellite. Anim Genet 24: 73. 10.1111/j.1365-2052.1993.tb00932.x 8498720

[28] Steffen P, Eggen A (1993) Isolation and mapping of polymorphic microsatellites in cattle. Anim Genet 24: 121–124. 8328693 10.1111/j.1365-2052.1993.tb00252.x

[29] Bishop MD, Kappes SM, Keele JW, Stone RT, Sunden SLF, et al (1994) A genetic linkage map for cattle. Genetics 136: 619–639. 7908653 10.1093/genetics/136.2.619 PMC1205813

[30] Moore SS, Byrne K (1994) Characterization of 65 bovine microsatellites. Mamm Genome 5: 84–90. 8180478 10.1007/BF00292333

[31] Carling MD, Passavant CW, Byers JA (2003) DNA microsatellites of pronghorn (*Antilocapra americana*). Mol Ecol Notes 3: 10–11.

[32] Thompson JD, Gibson TJ, Plewniak F, Jeanmougin F, Higgins DG (1997) The Clustal X windows interface: flexible strategies for multiple sequence alignment aided by quality analysis tools. Nucl Acids Res 25: 4876–4882. 9396791 10.1093/nar/25.24.4876 PMC147148

[33] Rozas J, Sánchez-DelBarrio JC, Messeguer X, Rozas R (2003) DnaSP, DNA polymorphism analyses by the coalescent and other methods. Bioinformatics 19: 2496–2497. 14668244 10.1093/bioinformatics/btg359

[34] Swofford DL (2001) PAUP*: phylogenetic analysis using parsimony (* and other methods), Version 4.0b8. Sunderland: Sinauer Associates.

[35] Felsenstein J (1985) Confidence limits on phylogenies: An approach using the bootstrap. *Evolution* 39: 783–791. 28561359 10.1111/j.1558-5646.1985.tb00420.x

[36] Drummond AJ, Rambaut A (2007a) BEAST: Bayesian evolutionary analysis by sampling trees. BMC Evol Bio 7: 214. 17996036 10.1186/1471-2148-7-214 PMC2247476

[37] Brown WM, George M Jr, Wilson AC (1979) Rapid evolution of animal mitochondrial DNA. Proc Natl Acad Sci USA 76: 1967–1971. 109836 10.1073/pnas.76.4.1967 PMC383514

[38] Aderson S, de Bruijn MH, Coulson AR, Eperon IC, Sanger F, et al (1982) Complete sequence of bovine mitochondrial DNA. Conserved features of the mammalian mitochondrial genome. J Mol Biol 156: 683–717. 7120390 10.1016/0022-2836(82)90137-1

[39] Meyer S, Weiss G, von Haeseler A (1999) Pattern of Nucleotide Substitution and Rate Heterogeneity in the Hypervariable Regions I and II of Human mtDNA. Genetics 152: 1103–1110. 10388828 10.1093/genetics/152.3.1103 PMC1460673

[40] Kass RE, Raftery AE (1995) Bayes factors. J Am Statist Assoc 90: 773–795.

[41] Rambaut A: FigTree: Tree figure drawing tool, version 1.0. Institute of Evolutionary Biology, University of Edinburgh, 2006.

[42] Posada D, Crandall KA (1998) MODELTEST: testing the model of DNA substitution. Bioinformatics 14: 817–818. 9918953 10.1093/bioinformatics/14.9.817

[43] Posada D, Crandall KA (2001) Intraspecific gene genealogies: trees grafting into networks. Trends Ecol Evol 16: 37–45. 11146143 10.1016/s0169-5347(00)02026-7

[44] Templeton AR, Crandall KA, Sing CF (1992) A cladistic analysis of phenotypic Associations with haplotypes inferred from restriction endonuclease mapping and DNA sequence data. III. Cladogram estimation. Genetics 132: 619–633. 1385266 10.1093/genetics/132.2.619 PMC1205162

[45] Clement M, Posada D, Crandall K (2000) TCS: a computer program to estimate gene genealogies. Mol Ecol 9: 1657–1659. 11050560 10.1046/j.1365-294x.2000.01020.x

[46] Schneider S, Kueffer JM, Roessli D, Excoffier L (2000) Arlequin ver. 2.000: A software for population genetics data analysis. Geneva (Switzerland): Genetics and Biometry Laboratory, University of Geneva.

[47] Excoffier L, Smouse PD, Quattro JM (1992) Analysis of molecular variance inferred from metric distances among DNA haplotypes: application to human mitochondrial DNA restriction data. Genetics 131: 479–494. 1644282 10.1093/genetics/131.2.479 PMC1205020

[48] Beerli P (2004) Migrate: documentation and program, part of LAMARC. Version 2.0. Distributed over the Internet. University of Washington website. Available: http://evolution.gs.washington.edu/lamarc.html. Accessed 2013 Mar 28.

[49] Beerli P, Felsenstein J (2001) Maximum likelihood estimation of a migration matrix and effective population sizes in subpopulations by using a coalescent approach. Proc Natl Acad Sci USA 98: 4563–4568. 11287657 10.1073/pnas.081068098 PMC31874

[50] Beerlin P, Felsenstein J (1999) Maximum-likelihood estimation of migration rates and effective population numbers in two populations using a coalescent approach. Genetics 152: 763–773. 10353916 10.1093/genetics/152.2.763 PMC1460627

[51] Drummond AJ, Rambaut A, Shapiro B, Pybus OG (2005) Bayesian Coalescent Inference of Past Population Dynamics from Molecular Sequences. Mol Bio Evol 22 (5): 1185–1192. 10.1093/molbev/msi103 15703244

[52] Rogers AR, Harpending HM (1992) Population growth makes waves in the distribution of pairwise genetic differences. Mol Bio Evol 9: 552–569. 1316531 10.1093/oxfordjournals.molbev.a040727

[53] Rogers AR (1995) Genetic evidence for a Pleistocene population explosion. Evolution 49: 608–615. 28565146 10.1111/j.1558-5646.1995.tb02297.x

[54] Slatkin M, Hudson RR (1991) Pairwise comparisons of mitochondrial DNA sequences in stable and exponentially growing populations. Genetics 129: 555–562. 1743491 10.1093/genetics/129.2.555 PMC1204643

[55] Fu Y, Li W (1993) Statistical tests of neutrality of mutations. Genetics 133: 693–709. 8454210 10.1093/genetics/133.3.693 PMC1205353

[56] Fu Y (1996) New statistical tests of neutrality for DNA samples from a population. Genetics 143: 550–557. 10.1093/genetics/143.1.557 PMC1207287 8722804

[57] Fu Y (1997) Statistical tests of neutrality against population growth, hitchhiking and background selection. Genetics 147: 915–925. 9335623 10.1093/genetics/147.2.915 PMC1208208

[58] Ramos-Onsins SE, Rozas J (2002) Statistical properties of new neutrality tests against population growth. Mol Bio Evol 19: 2092–2100. 12446801 10.1093/oxfordjournals.molbev.a004034

[59] Jensen JL, Bohonak AJ, Kelley ST (2005) Isolation by distance, web service. BMC Genetics. 6: 13. 10.1186/1471-2156-6-13 PMC1079815 15760479

[60] Goudet J (1995) Fstat: a computer program to test F-statistics. J Hered 86: 485–486.

[61] Raymond M, Rousset F (1995) An exact test for population differentiation. Evolution 49: 1280–1283. 28568523 10.1111/j.1558-5646.1995.tb04456.x

[62] Pritchard JK, Stephens M, Donnelly P (2000) Inference of population structure using multilocus genotype data. Genetics 155: 945–959. 10835412 10.1093/genetics/155.2.945 PMC1461096

[63] Evanno G, Regnaut S, Doudet J (2005) Detecting the number of clusters of individuals using the software STRUCTURE: a simulation study. Mol Ecol 14: 2611–2620. 15969739 10.1111/j.1365-294X.2005.02553.x

[64] Rosenberg N (2004) DISTRUCT: a program for the graphical display of population structure. Mol Ecol Notes 4: 137–138.

[65] Cornuet JM, Luikart G (1996) Description and power analysis of two tests for detecting recent population bottlenecks from allele frequency data. Genetics 144: 2001–2014. 8978083 10.1093/genetics/144.4.2001 PMC1207747

[66] Luikart G, Allendorf FW, Cornuet JM (1997) William B Sherwin (1997) Distortion of allele frequency distributions provides a test for recent population bottlenecks. J Hered 89: 238–247. 10.1093/jhered/89.3.238 9656466

[67] Luikart G, Cornuet JM (1998) Empirical evaluation of a test for identifying recently bottlenecked populations from allele frequency data. Conserv Biol 12: 228–237.

[68] Morin PA, Moore JJ, Chakraorty R, Jin L, Goodall J, et al (1994) Kin selection, social structure, geneflow, and the evolution of chimpanzees. Science 265: 1193–1201. 7915048 10.1126/science.7915048

[69] Ferris SD, Sage RD, Huang CM, Nielsen JT, Ritte U, et al (1993) Flow of mitochondrial DNA across a species boundary. Proc Natl Acad Sci U S A 80: 2290–2294. 10.1073/pnas.80.8.2290 PMC393805 6300907

[70] Avise JC, Bermingham E, Kessler LG, Saunders NC (1984) Characterization of mitochondrial DNA variability in a hybrid swarm between subspecies of bluegill sunfish (*Lepomis macrochirus*). Evolution 38: 931–941. 28555798 10.1111/j.1558-5646.1984.tb00364.x

[71] An Z, Kutzbach JE, Prell WL, Porter SC (2001) Evolution of Asian monsoons and phased uplift of the Himalaya-Tibetan Plateau since Late Miocene times. Nature 411: 62–66. 11333976 10.1038/35075035

[72] Qiu Q, Zhang G, Ma T, Qian W, Wang J, et al (2012) The yak genome and adaptation to life at high altitude. *Nature Genet* 44: 946–949. 22751099 10.1038/ng.2343

[73] Pruett CL, Saillant E, Gold JR (2005) Historical population demography of red snapper (*Lutjanus campechanus*) from the northern Gulf of Mexico based on analysis of sequences of mitochondrial DNA. Mar Biol 147: 593–602.

[74] Gold JR, Pak E, Richardson LR (2001) Microsatellite variation among red snapper (*Lutjanus campechanus*) from the Gulf of Mexico. J Mar Biotechnol 3: 293–304. 10.1007/s10126-001-0004-7 14961368

[75] Patterson WF, Watterson JC, Shipp RL, Cowan JH (2001) Movement of tagged red snapper in the northern Gulf of Mexico. T Am Fish Soc 130: 533–545.

[76] Schaller GB, Ren J, Qiu M (1991) Observations on the Tibetan antelope (*Pantholops hodgsonii*). Appl Anim Behav Sci 29: 361–378.

[77] Ginsberg GR, Schaller GB, Lowe J (2000) A petition to list the Tibetan antelope (*Pantholops hodgsonii*) as an endangered species pursuant to the U.S. Endangered Species Act of 1973. Wildlife Conservation Society & Tibetan Plateau Project petition to list Tibetan antelope as an endangered species. Geneva, Switzerland, CITES Secretariat.

[78] Schaller GB, Kang A, Cai I, Liu Y (2006) Migratory and calving behavior of Tibetan antelope population. Acta Theriol Sinica 26: 105–113.

[79] Hewitt G (2004) Genetic consequences of climatic oscillations in the Quaternary. Philo T Roy Soc B 359: 183–195. 10.1098/rstb.2003.1388 PMC1693318 15101575

[80] Shapiro B, Drummond AJ, Rambaut A, Wilson MC, Matheus PE, et al (2004) Rise and fall of the Beringian steppe bison. Science 306: 1561–1565. 15567864 10.1126/science.1101074

[81] Stiller M, Baryshnikov G, Bocherens H, d'Anglade AG, Hilpert B, et al (2010) Withering Away-25,000 Years of Genetic Decline Preceded Cave Bear Extinction. Mol Biol Evol 27: 975–978. 20335279 10.1093/molbev/msq083

[82] Miller DJ (1999) Nomads of the Tibetan Plateau rangelands in Western China part three: pastoral development and future challenges. Rangelands 21: 17–20.

[83] Templeton AR, Robertson RJ, Brisson J, Strasburg J (2001) Disrupting evolutionary processes: The effect of habitat fragmentation on collared lizards in the Missouri Ozarks. Proc Natl Acad Sci USA 98: 5426–5432. 11344289 10.1073/pnas.091093098 PMC33229

[84] Yang J, Jiang Z (2011) Genetic diversity, population genetic structure and demographic history of Przewalski's gazelle (Procapra przewalskii): Implications for conservation. Conserv Genet 12: 1457–1468.

[85] Avise JC, Arnold J, Ball RM, Bermingham E, Lamb T, et al (1987) Intraspecific phylogeography: the mitochondrial DNA bridge between population genetics and systematics. Annu Rev Ecol Evol 18: 489–522.

[86] Russell AL, Medellin RA, Maccracken GF (2005) Genetic variation and migration in the Mexican free-tailed bat (*Tadarida brasiliensis mexicana*). Mol Ecol 14: 2207–2222. 15910338 10.1111/j.1365-294X.2005.02552.x

